# Rapid thermal annealing for high-quality ITO thin films deposited by radio-frequency magnetron sputtering

**DOI:** 10.3762/bjnano.10.149

**Published:** 2019-07-25

**Authors:** Petronela Prepelita, Ionel Stavarache, Doina Craciun, Florin Garoi, Catalin Negrila, Beatrice Gabriela Sbarcea, Valentin Craciun

**Affiliations:** 1 National Institute for Laser, Plasma and Radiation Physics, P.O. Box MG-36, Magurele 077125, Ilfov, Romania; 2 National Institute of Materials Physics, 405A Atomistilor Street, P.O. Box MG-7, Magurele 077125, Ilfov, Romania; 3 ICPE-CA, Splaiul Unirii 313, Sector 3, 74204, Bucharest, Romania; 4 Dentix MILLENNIUM SRL, Sabareni-Ilfov, Romania

**Keywords:** conductive transparent electrodes, indium tin oxide (ITO) films, optical properties, radio-frequency magnetron sputtering (rfMS), rapid thermal annealing (RTA)

## Abstract

In this work, rapid thermal annealing (RTA) was applied to indium tin oxide (ITO) films in ambient atmosphere, resulting in significant improvements of the quality of the ITO films that are commonly used as conductive transparent electrodes for photovoltaic structures. Starting from a single sintered target (purity 99.95%), ITO thin films of predefined thickness (230 nm, 300 nm and 370 nm) were deposited at room temperature by radio-frequency magnetron sputtering (rfMS). After deposition, the films were subjected to a RTA process at 575 °C (heating rate 20 °C/s), maintained at this temperature for 10 minutes, then cooled down to room temperature at a rate of 20 °C/s. The film structure was modified by changing the deposition thickness or the RTA process. X-ray diffraction investigations revealed a cubic nanocrystalline structure for the as-deposited ITO films. After RTA, polycrystalline compounds with a textured (222) plane were observed. X-ray photon spectroscopy was used to confirm the beneficial effect of the RTA treatment on the ITO chemical composition. Using a Tauc plot, values of the optical band gap ranging from 3.17 to 3.67 eV were estimated. These values depend on the heat treatment and the thickness of the sample. Highly conductive indium tin oxide thin films (ρ = 7.4 × 10^−5^ Ω cm) were obtained after RTA treatment in an open atmosphere. Such films could be used to manufacture transparent contact electrodes for solar cells.

## Introduction

Thin oxide films, used as contact electrodes [1–4], are considered to be important components of photovoltaic cells [5–6]. As an electrode candidate for solar cells, an ITO film [7–8] must present excellent optical and electrical properties for increased energy generation. At this time, the goal is to obtain high-performance solar cells [6,9–10] but at a cost as low as possible. ITO is an attractive material because it exhibits excellent properties as a transparent conductive oxide (TCO) material that can be tuned during deposition in order to obtain materials for specific applications [7,11]. Due to these features, ITO films are promising components for the development of high-performance optoelectronics [7,11–13] and photovoltaic devices. In order to use ITO thin films for photovoltaic applications, samples with reproducible properties are required [8,14]. The performance of ITO in various applications increases when the electrical properties are improved.

Various deposition techniques have been used to obtain TCO thin films, such as: vacuum thermal evaporation [15–16], chemical vapor deposition [17], sol–gel [18], pyrolysis spray techniques [5,19], magnetron sputtering [20–22], and pulsed laser deposition (PLD) [23].

Various changes in the optical, electrical and structural qualities are known to occur depending on the type of deposition and thermal treatment process [24–30] applied to the ITO films. For instance, an improvement in the optical transmission by 52.8% (from 34.2% to 87% at 550 nm) due to heat treatment at 850 °C in nitrogen atmosphere was reported [24]. Thermal treatments become important when the application of ITO films on a large scale can be demonstrated with excellent performance for various applications [7–10].

Despite its low cost, the radio-frequency magnetron sputtering (rfMS) deposition technique [31–32] involves sophisticated deposition equipment, allowing for the quick production of functional coatings, in addition to the possibility of adjusting the deposition parameters of the thin films to provide a desired structure.

In this paper, the influence of rapid thermal annealing (RTA) on the structure and optical properties of ITO films, obtained by rfMS, are reported. We conducted studies to optimize the deposition parameters in order to obtain ITO thin films with excellent properties. The main accomplishment of this work consists in obtaining ITO films of high quality, that is, good uniformity, high transparency (93%), and good conductivity (1.35 × 10^4^ Ω^−1^ cm^−1^). The novelty of this study lies in the RTA process applied to the ITO films, which exhibited a beneficial influence on the structural, electrical and optical properties, making them highly suitable for use as efficient electrodes in photovoltaic devices.

## Experimental

Indium tin oxide (ITO) films with thicknesses ranging from 230 to 370 nm were deposited by radio-frequency magnetron sputtering (rfMS). Quartz substrates (commercially available from Neyco) with a size of 20 × 20 × 1 mm, with both sides polished, were used for the deposition of ITO thin films. An ITO commercial target (KJLC, 4" diameter, 0.125" thick) was mounted at the planar cathode of the magnetron. The deposition of the ITO films was achieved with a vacuum system (a mechanical pump and a cryo pump) and a complementary vacuum system, respectively, consisting of a mechanical pump and a turbo-molecular pump, to achieve a high vacuum in the reaction chamber, which has a volume of 0.2 m^3^.

Before deposition, the chamber was pumped down until the pressure reached 2.67 × 10^−4^ Pa. Subsequently, the working gases (i.e., high purity argon and oxygen) were introduced into the deposition chamber, and a power of 70 W was applied to the magnetron.

The ITO thin films were obtained at room temperature at a pressure of 6.67 × 10^−1^ Pa and a deposition rate of 9.6 nm/min. The deposition pressure, argon and oxygen flow rates and the applied electrical current were kept constant during each deposition. More specifically, the deposition parameters and the studied sample characteristics are summarized in Table 1.

**Table 1:** Parameters and conditions for the production of ITO thin films.

Parameters	Conditions

target	ITO (In_2_O_3_/SnO_2_ 90/10 w/w)
aubstrate	Quartz
ITO film thickness (nm)	230, 300, 370
substrate to target distance (mm)	90
sputtering pressure before deposition (Pa)	2.67 × 10^−4^
sputtering pressure during deposition (Pa)	6.67 × 10^−1^
flow rate of Ar 99.999% (sccm)	30
flow rate of O_2_ 99.999% (sccm)	1.5
deposition rate (nm min^−1^)	9.6
electrical current (A)	0.1
forward power (W)	70
reflected power (W)	0
annealing temperature (°C)	575

The predefined thickness values (230 nm, 300 nm, 370 nm) for the ITO samples were measured with a thickness monitor, provided by a quartz crystal as a basic transducer. The crystal is excited in mechanical motion with an external oscillator. When the film reaches the preprogrammed thickness value, the shutter located above the target automatically closes.

After deposition, the ITO films were subjected to RTA in air. This process was performed in an open resistive furnace (MILA 5000) in order to improve the structural and optical properties of the films. Thus, the samples were subjected to 575 °C at a heating rate of 20 °C/s and maintained at the thermal threshold for 10 min. Then, they were cooled down to room temperature at a rate of 20 °C/s. Time control of the RTA process allowed us to avoid exfoliation of the film from the substrate.

The thickness of the films was also experimentally verified after deposition using interference microscopy in order to verify the predefined thickness values. Scanning electron microscopy (SEM) investigations (FEI Quanta) were performed to observe the structures and morphology of the deposited surfaces. Given that the thickness of the quartz substrate is much greater than that of the ITO conductive film, a thin layer of gold was sputtered onto the sample surfaces prior to imaging and cross-sectional analysis to avoid electrostatic charging during the measurements.

The elemental and chemical composition of the ITO thin films as well as the electronic state of the elements within the material were investigated by X-ray photoelectron spectroscopy (XPS) method. A SPECS spectrometer with a PHOIBOS RX 150 analyzer and a Specs XR–50 M source was operated with a monochromatic Al anode (*h*ν = 1486.61 eV) at 300 W. The charging effect of the sample deposited onto the quartz substrate is compensated for with a Specs FG15/40 flood gun. The chemical composition values of the component elements were determined from the XPS spectra using the Avantage software (version 5.978). The crystalline structure of the ITO thin films on amorphous quartz substrates was investigated by grazing incidence X-ray diffraction (XRD, Bruker AXS D8 Discover diffractometer) with Cu Kα radiation (λ = 1.5418 Å) at a power of 40 kV/40 mA. The scan was performed between 20° and 60° with a 2 s/step scan speed and 0.04° step.

Optical transmission spectra of the deposited thin films were measured with a UV–vis–NIR Perkin Elmer 950 spectrophotometer (190–3300 nm). The electrical conductivity values were measured using a two-channel B2901A measurement unit (KEYSIGHT technology).

## Results and Discussion

**Figure 1:**
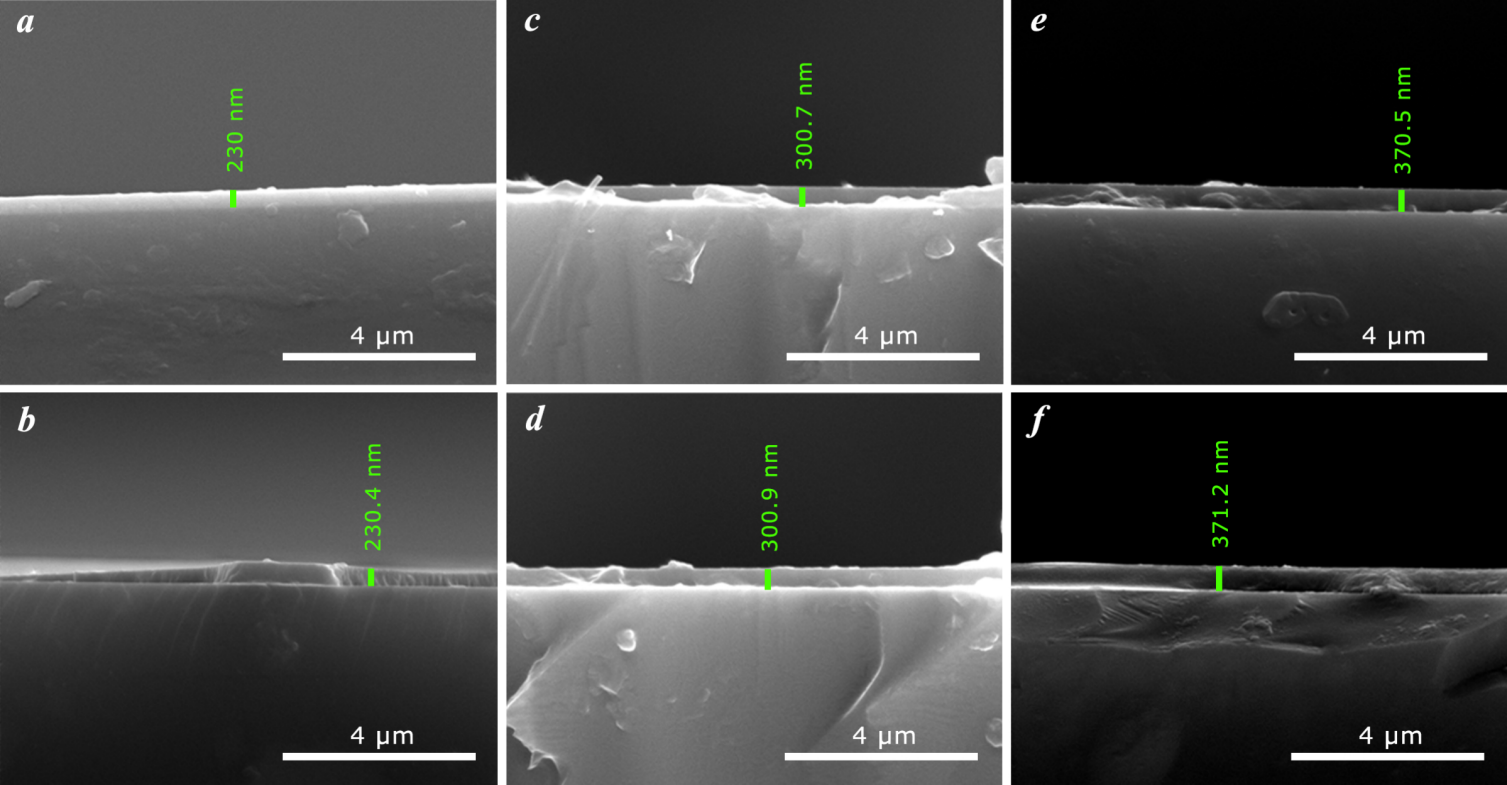
Cross-sectional SEM images of the cleaved as-deposited (a,c,e) and RTA-processed (b,d,f) ITO films, with measured thickness values of: a) *d*_ITO230_ = 230 nm, b) *d*_ITO230_ = 230.4 nm; c) *d*_ITO300_ = 300.7 nm, d) *d*_ITO300_ = 300.9 nm, e) *d*_ITO370_ = 370.5 nm, and f) *d*_ITO370_ = 371.2 nm.

The analysis of the morphology of the nanostructured surface of both the as-deposited and RTA-processed ITO thin films was conducted using SEM. This method allows for the layer thickness to be measured from cross-sectional analysis with SEM (Figure 1). The measured values are in good agreement with the predefined ones (±1.2 nm).

**Figure 2:**
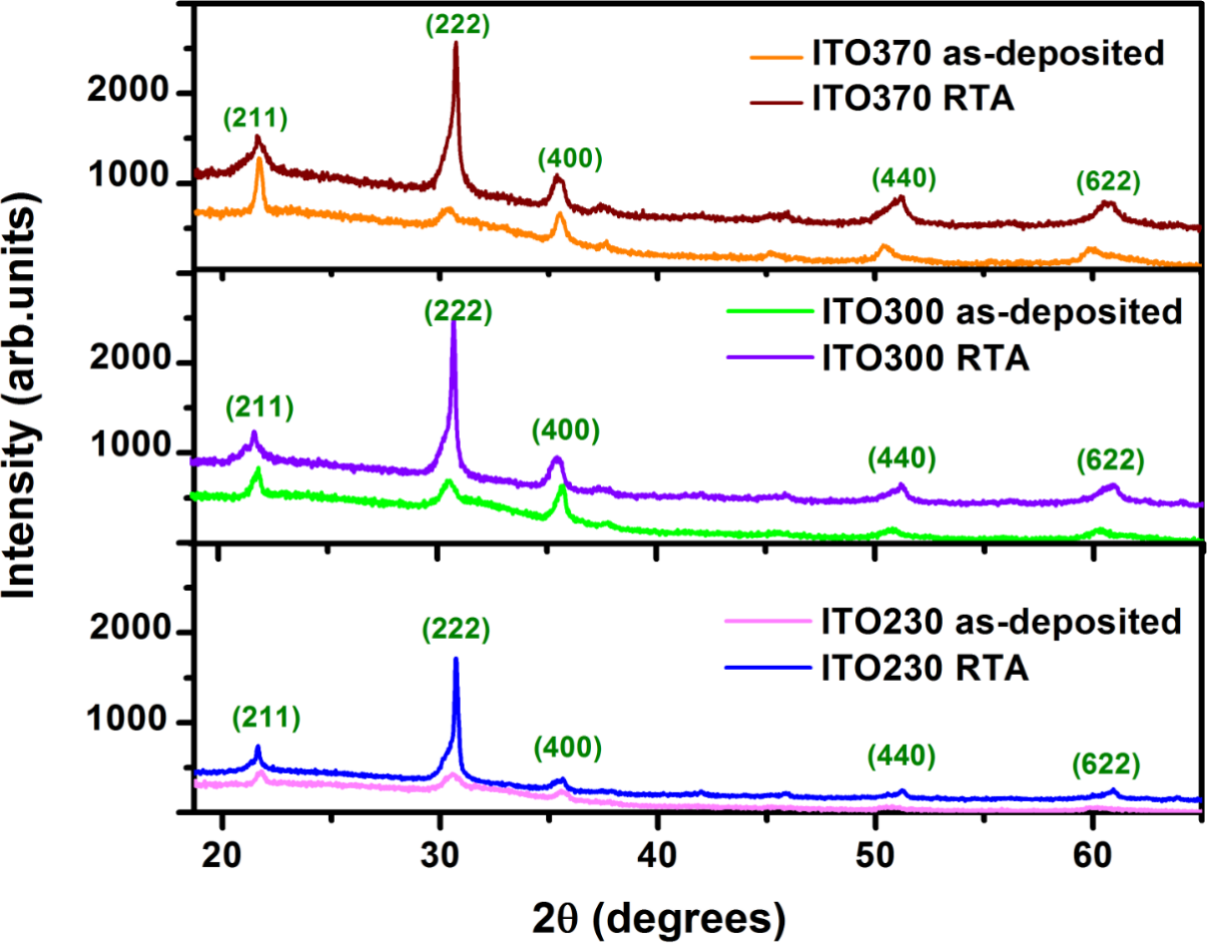
XRD patterns for the as-deposited and RTA-processed ITO samples.

The uniformity of the as-deposited thin films, as observed by the SEM measurements (Figure 1a,c,e), is due to the optimal deposition conditions, namely, a low deposition rate and adjustment of the magnetron power for current intensities as small as possible. As a result of RTA processing, layers with strong adhesion to the substrate are achieved (see Figure 1b,d,f). At a specific temperature applied to the deposited samples, the elimination of gaseous impurities from degassing of the deposition chamber takes place. At the same time, the improvement of surface crystallinity (Figure 2), annealing of surface structural defects and achievement of uniform films on the entire deposited surface (Figure 1) are obtained [33].

Using these structural and morphological techniques to analyze the ITO thin films, we could report improvements in film quality following the RTA treatment. These properties are clearly evidenced by XRD in Figure 2. The investigated XRD patterns for the as-deposited ITO films showed a cubic nanocrystalline structure. Due to the high temperature used during RTA, the films became polycrystalline. An increase of the peak intensity with the increase in film thickness to 370 nm can also be noticed (Figure 2), as well as a structural reorientation (decrease of the main peak intensity and the appearance of other peaks in the film structure) compared to the lower thickness sample (e.g., 230 nm).

The lattice constant for the cubic structure of the ITO thin films was determined according to [34–36]:


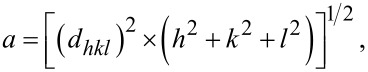


where *d*_hkl_ is the interplanar spacing and *h*, *k*, *l* are the Miller indices.

According to standard ASTM (i.e., American Society for Testing Materials) diffraction charts [36], the cell parameter has the value *a* = 10.130 (Å) while the computed parameters are *a* = 10.144 (Å), for the ITO sample of 230 nm thickness. It is also found that there is a displacement of the peaks to smaller angles after RTA treatment, which leads to an increase in the cell parameters. Accordingly, the crystallite size of the RTA-treated sample becomes 12.68 nm perpendicular to the direction of the plane (222), 18.13 nm for the plane (400), and 11.87 nm for the plane (440). The RTA of ITO films proved to be a key step in improving their performance, as indicated by the increase in the crystallite size (Table 2) and the reduction of structural defects.

**Table 2:** XRD results for as-deposited and RTA-treated ITO thin films, with different thickness values.^a^

*d* (nm)	Sample	(*hkl*)	2θ (degrees)	β_2θ_ (mrad)	*D* (nm)	*d*_hkl_ (Å)	*a* (Å)

230	ITO as-deposited	(222)	30.80	11.34	12.68	2.902	10.144
		(400)	35.44	8.02	18.13	2.532	
		(440)	50.52	12.91	11.87	1.786	
	ITO RTA-treated	(222)	30.80	4.19	34.35	2.902	10.130
		(400)	35.44	8.20	17.75	2.532	
		(440)	51.10	5.06	30.37	1.805	
300	ITO as-deposited	(222)	30.73	10.64	13.51	2.908	10.147
		(400)	35.60	6.28	23.18	2.524	
		(440)	50.89	14.65	10.47	1.783	
	ITO RTA-treated	(222)	30.73	4.19	34.34	2.908	10.132
		(400)	35.55	9.59	15.17	2.521	
		(440)	51.19	10.29	14.95	1.793	
370	ITO as-deposited	(222)	30.70	4.88	29.43	2.911	10.150
		(400)	35.55	9.77	14.90	2.524	
		(440)	51.22	13.78	11.15	1.783	
	ITO RTA-treated	(222)	30.50	9.94	34.45	2.930	10.131
		(400)	35.55	7.50	19.41	2.524	
		(440)	51.22	8.37	18.36	1.783	

^a^Thin film thickness, (*hkl*) – Miller indices corresponding to diffraction planes, θ – Bragg angle, β – half-width of the diffraction peak, *D* – size of the crystallites [34–35], *d*_hkl_ – interplanar spacing, *a* – crystal lattice constant; with λ = 1.54 Å is the wavelength of the incident radiation and *D* is the crystallite size perpendicular to the plane corresponding to the measured diffraction peak.

**Figure 3:**
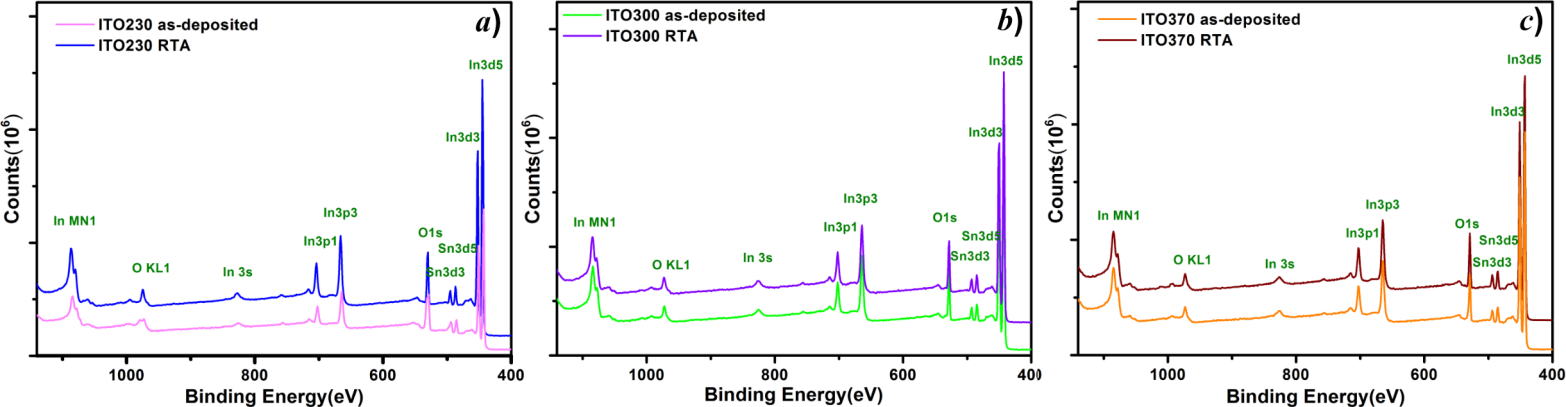
XPS survey spectra in the range 0–1250 eV acquired from the as-deposited and RTA-treated ITO samples of thickness a) 230 nm, b) 300 nm, and c) 370 nm.

To evaluate the chemical composition of the surface for the as-deposited and RTA-processed films, the XPS spectra were recorded, as shown in Figure 3a–c. The spectra were recorded over a wide range of binding energies (0–1150 eV), which revealed the presence of all the intended elements on the surface. The data was calibrated relative to the standard peak of adventitious C 1s with an energy of 284.8 eV. The binding energies of the XPS core peaks (O 1s, In 3d, and Sn 3d), for the as-deposited and RTA-treated samples are presented in Table 3 together with the relative atomic concentrations.

**Table 3:** Typical chemical composition of ITO thin films, determined by XPS measurements.

ITO thin films	*d* (nm)	O (atom %)	In (atom %)	Sn (atom %)

as-deposited	230	54.8	41.1	4.1
RTA-treated	230	65.2	31.7	3.1
as-deposited	300	55.5	40.5	4.0
RTA-treated	300	62.3	34.3	3.4
as-deposited	370	63.2	33.5	3.3
RTA-treated	370	61.3	35.2	3.5

**Figure 4:**
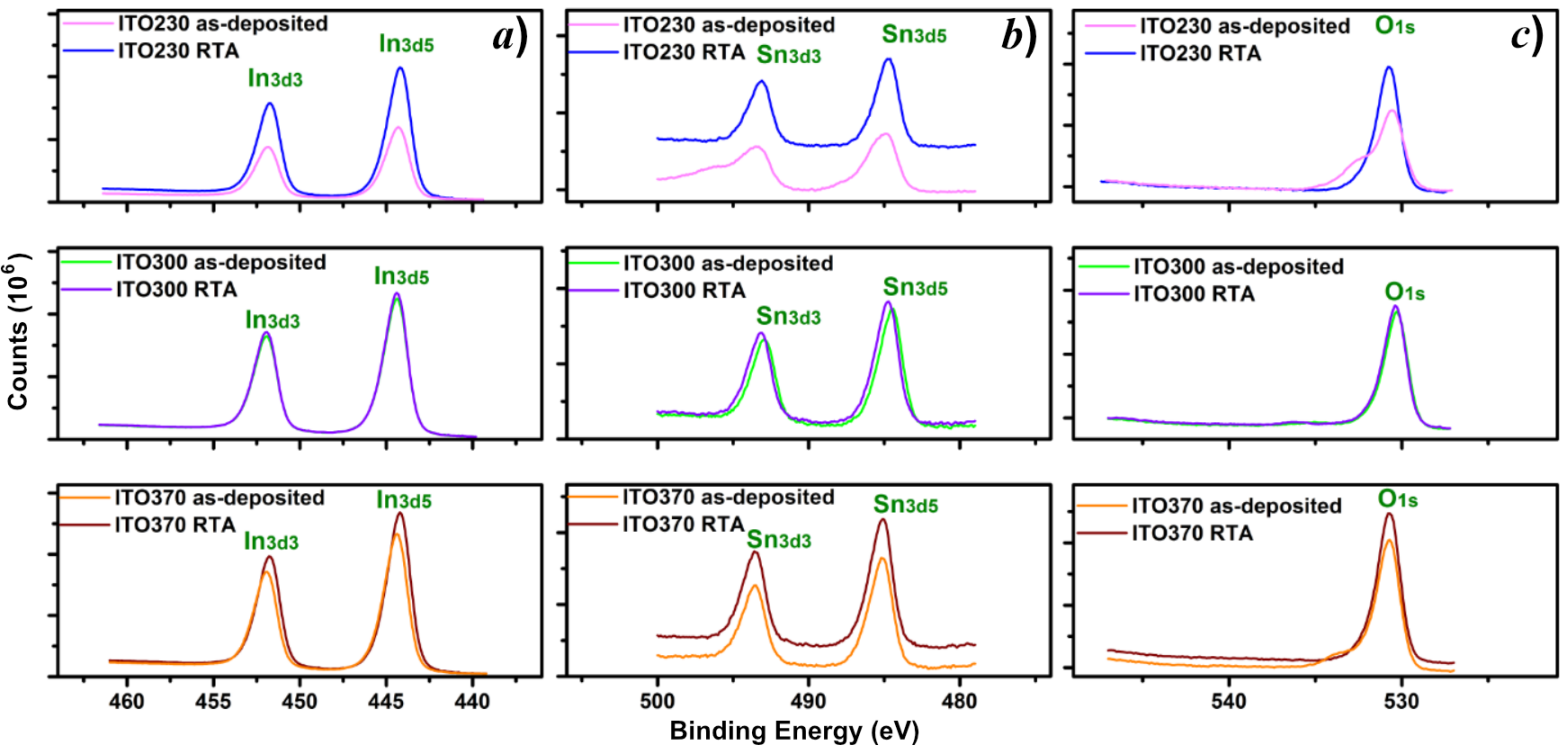
High-resolution a) In 3d, b) Sn 3d and c) O 1s XPS spectra acquired from as-deposited and RTA-treated ITO samples of thickness 230 nm, 300 nm and 370 nm.

The high-resolution spectra of these core level transitions are also depicted in Figure 4a (In 3d), Figure 4b (Sn 3d) and Figure 4c (O 1s) [37–38]. The binding energy of the thermally treated samples is slightly shifted toward a lower value, which is most likely due to changes in morphology of the samples. There are structural changes induced by incorporation of SnO_2_ into the In_2_O_3_ matrix, most probably by substitution of In ions with Sn and formation of the ITO compound. Noteworthy, this treatment also favors a low percentage of bound surface oxygen, which could lead to an improvement in sensitivity of the ITO film. The samples are made up of ITO (in overwhelming proportion) and probably islands of In and Sn independent oxides (Figure 4a–c). The amount of the latter decreases for the treated samples. These phases exhibited a different electrical charge, so it is reasonable to assume that the associated binding energies are slightly higher. The amount of oxides decreases in the treated samples as compared to untreated ones (Table 3). Separate phases of In_2_O_3_ and SnO_2_, respectively, are observed in all studied samples. The In_2_O_3_ phase appears in a larger quantity for the untreated samples and decreases with increasing thickness (230–370 nm) of the layer for both treated and untreated samples. On the other hand, the amount of Sn oxides decreases for the treated samples. Therefore, it can be concluded that ITO is very stable on the surface of the samples after the application of RTA. Note that XRD did not reveal the presence of a characteristic phase for In_2_O_3_ or SnO_2_, but only the ITO phase.

The study of the optical properties and, in particular, the absorption of light in transparent ultra-thin oxide films is of practical (the manufacturing and operation of various optical and optoelectronic devices) and theoretical (the structure of energy bands, local levels, material purity, determination of optical constants, etc.) importance. An in-depth understanding of the optical properties of as-deposited and thermally treated ITO films is essential in assessing the advantages of the RTA procedure.

**Figure 5:**
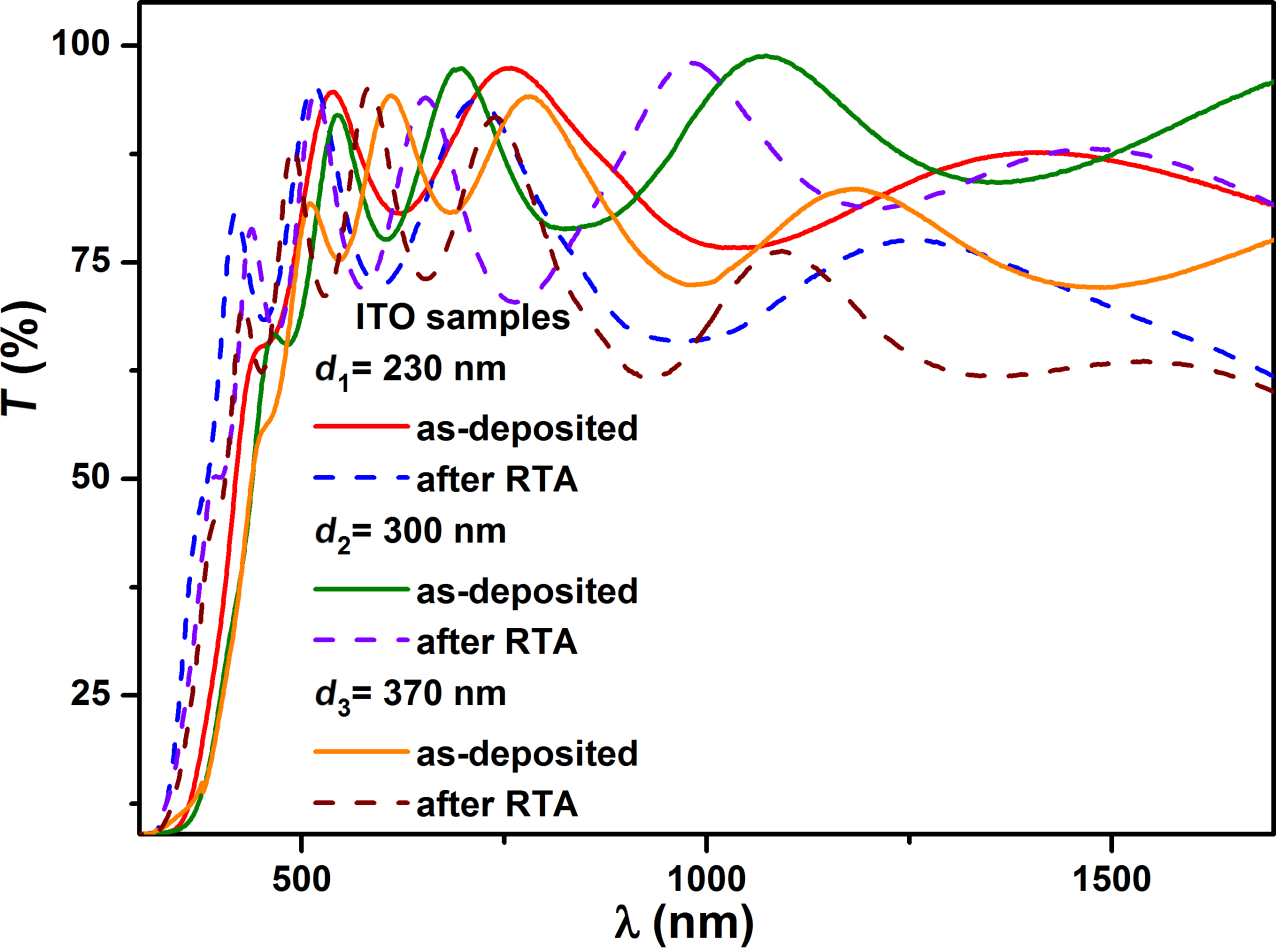
Optical transmission spectra for the as-deposited and RTA-treated ITO samples with interference maxima and minima for film thickness of 230 nm, 300 nm, and 370 nm.

To obtain information on the bandgap width, absorption coefficient, refractive index, extinction coefficient, dielectric permittivity, position of the impurity levels in the bandgap and characteristics of the optical transitions, we plotted and studied the transmission spectra of the ITO thin films (Figure 5). These polycrystalline thin films show considerable promise for integration into photovoltaic structures, having the bandgap width close to the optimum value of 3.7 eV [39]. Following the RTA process, the ITO thin films achieve significantly improved performance, both structurally and optically. The effect of RTA and the various deposition conditions were analyzed using the Swanepoel [40] and Drude [41–43] methods, in order to obtain the optical constants.

Spectral analysis in the UV–vis–NIR range of these oxide films (Figure 5) was described by the free electron model. Thus, an algorithm using computational models and the optical properties for oxide films (Swanepoel and Wemple–DiDomenico models [44]) was developed.

The presence of interference maxima and minima in the transmission spectra of ITO thin films (84–93%) (Figure 5) allowed the determination of their optical constants by using the envelope method (Swanepoel).

The optical constants were calculated for strong and low absorption domains. It is noted that optical absorption is significant in the 400–1000 nm spectral range, and the edge of the absorption band moves toward higher wavelengths as the thickness of the layers increases. The stronger absorption of layers after rapid annealing is influenced by the increased volume of intercrystalline regions. In the literature it is reported that optical absorption is strong in the intercrystalline region. Thus, increasing the volume of the intercrystalline regions together with the rapid heating of the layer leads to overall increased optical absorption.

**Figure 6:**
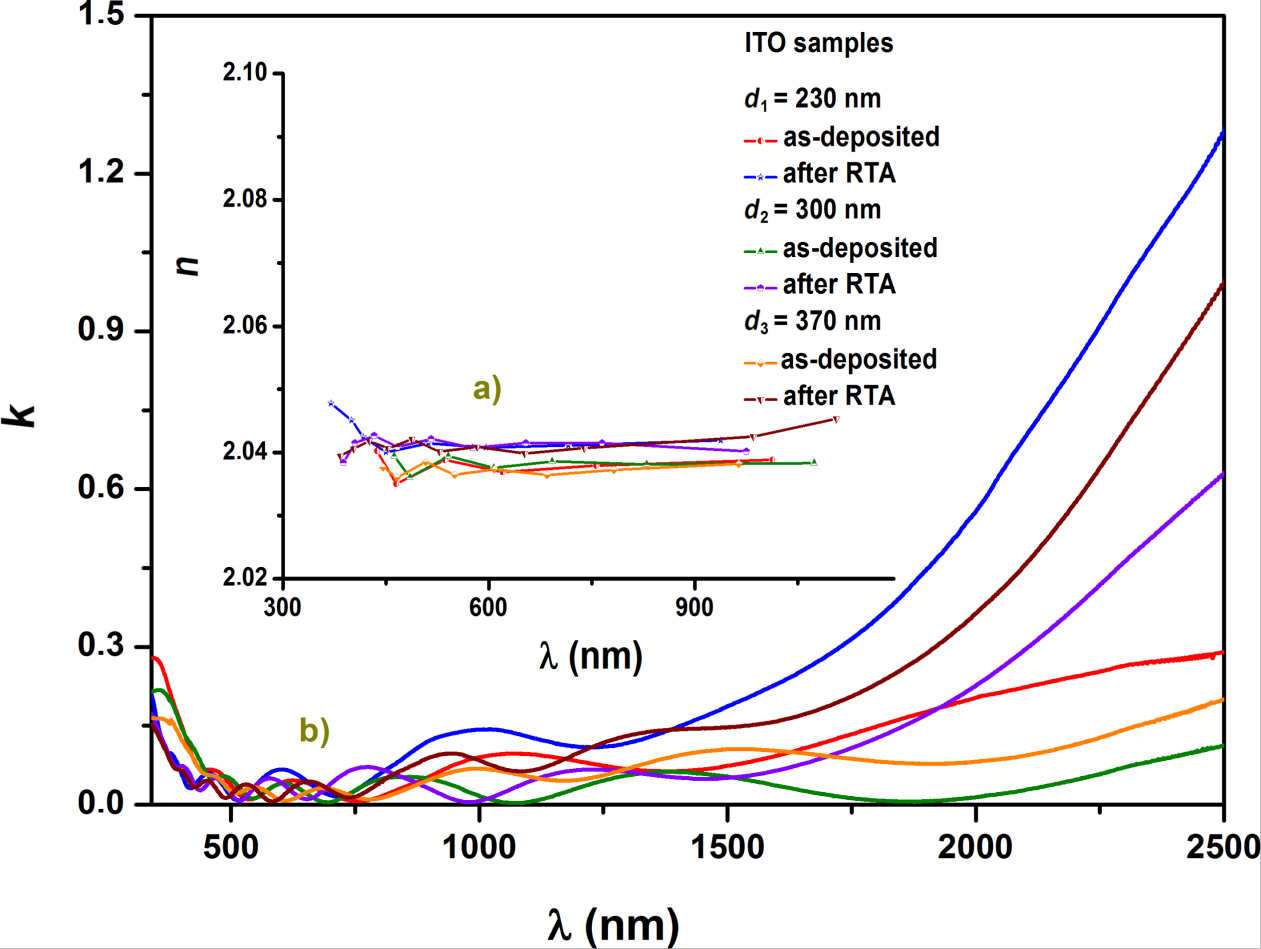
a) Refractive index dependence on the wavelength (dispersion) and b) extinction coefficient dependence on the wavelength for the as-deposited and RTA-treated ITO samples.

In the transmission spectra (400–1000 nm), the two envelopes *T*_M_ and *T*_m_ corresponding to interference maxima and minima were plotted, and the values of the refractive index, *n,* (Figure 6a) of the films were determined according to the formulas shown below. In the case of medium and weak absorption domains, one obtains [40,45–46]:

[1]
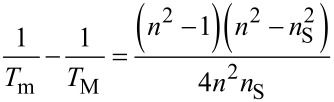


where the refractive index is written as [40,43]:

[2]
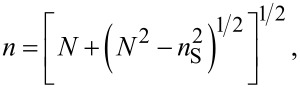


where

[3]
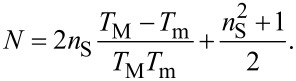


The extinction coefficient dependence on the wavelength (250–2500 nm) of the investigated samples is presented in Figure 6b. A normal dispersion in the spectral range of measurements is noted. By computing the average value of the refractive index over the investigated wavelength range, it is found that in general the refractive index of the layer increases with thickness. From the graphs it is inferred that at a wavelength of 500 nm the refractive index of the layers approaches the value of the ITO target. The dispersion of the refractive index can be described using the single oscillator model and was expressed by Wemple and DiDomenico using the average energy of the oscillator, *E*_0_, and dispersion energy, *E*_d_ [44] as:

[4]
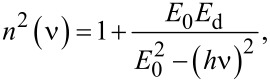


where ν is the photon frequency. From the graphical representation (*n*^2^ − 1)^−1^ = *f* [(*h*ν)^2^] we get the slope (*E*_0_*E*_d_)^−1^, where *E*_0_ is considered to be an average of the bandgap energy of semiconductor and has the expression *E*_0_ ≈ 2*E*_g_. The optical width of the bandgap can be determined from the study of fundamental (intrinsic) absorption in thin layers.

**Figure 7:**
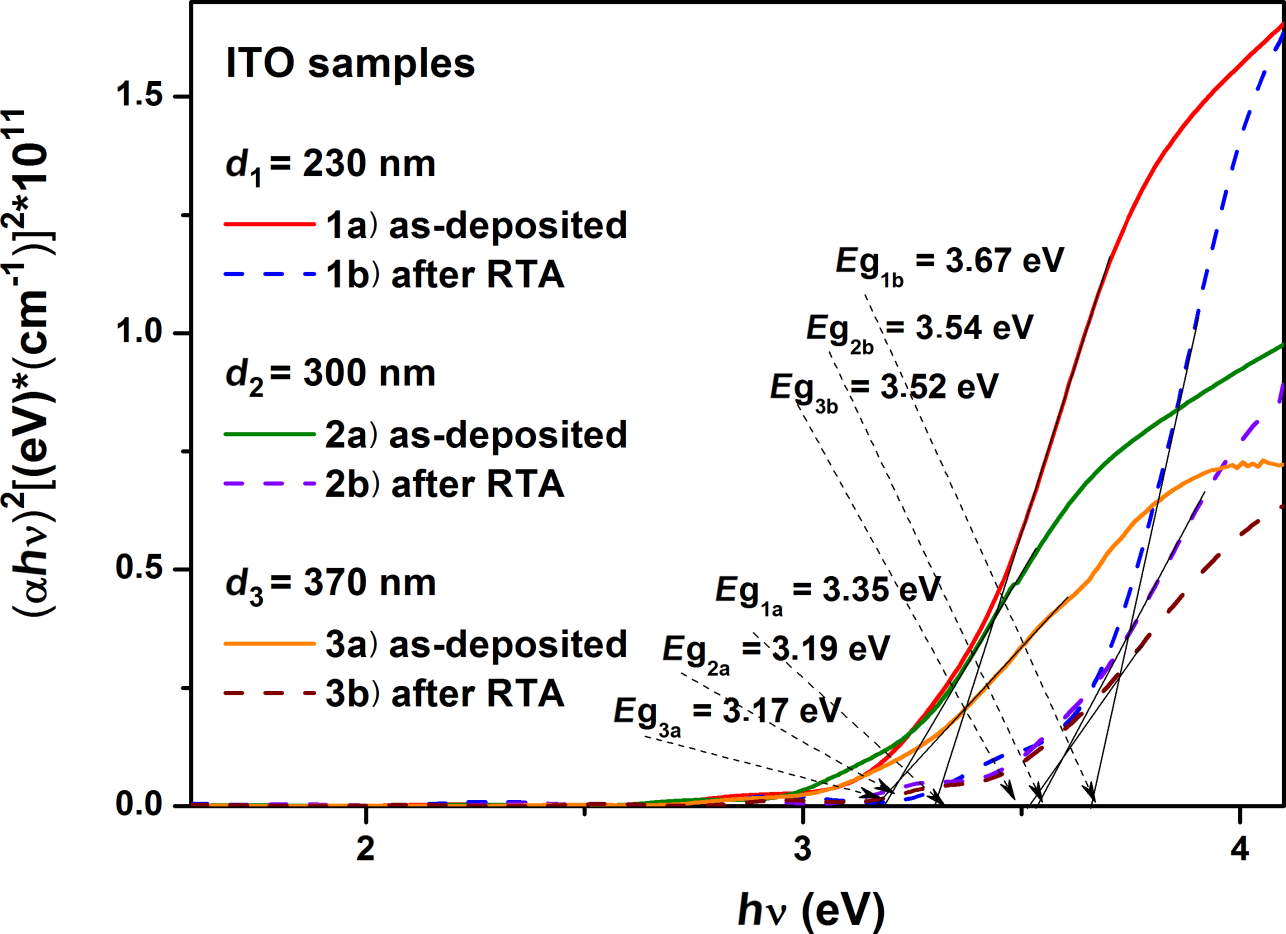
Dependence of (α*h*ν)^2^ = *f*(*h*ν) on the energy of the incident photons, for the as-deposited and RTA-treated ITO samples.

We determined the optical width of the bandgap corresponding to the direct optical transitions, 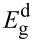, by extrapolating the linear portion of the dependence (α*h*ν)^2^ = *f*(*h*ν) to (α*h*ν)^2^ → 0. Using a Tauc plot, the bandgap width for the ITO films was determined to be between 3.17 eV and 3.67 eV (Figure 7). The values of the bandgap width corresponding to direct optical transitions, determined from these dependencies, are shown in Table 4.

**Table 4:** The values of the bandgap width corresponding to direct optical transitions and the electrical conductivity for the as-deposited and RTA-treated ITO samples.^a^

ITO thin film	*d* (nm)	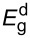 (eV)	σ (Ω^−1^ cm^−1^) × 10^4^

as-deposited	230	3.35	0.52
RTA-treated	230	3.67	1.12
as-deposited	300	3.19	0.55
RTA-treated	300	3.54	1.25
as-deposited	370	3.17	0.58
RTA-treated	370	3.52	1.35

^a^*d* is thin film thickness, 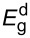 is the optical width of the bandgap corresponding to the direct optical transitions and σ is the electrical conductivity.

In agreement with other researchers [28–29] regarding ITO films deposited by rfMS, the thermal treatments show superior performance in terms of the optical properties, such as an increase of the bandgap by 0.41 eV. On the other hand, ITO films deposited by electron beam evaporation do not exhibit an improvement of their optical properties even after thermal treatment. On the contrary, they show a decrease of the bandgap by 0.52 eV [26]. An explanation could be the type of deposition that is used and the way the thermal treatment is performed. This aspect was mentioned [28], and the ITO thin films are strongly influenced structurally by the deposition technique and applied thermal treatment conditions, resulting in the improved optical and electrical properties.

For all sets of samples, it was found that the bandgap width corresponding to direct transitions has values similar to those reported in the literature [39], that is, they decrease with increasing thickness of the thin films. This leads to a reduction of the intervals between them and, consequently, of the bandgap. The width of the bandgap corresponding to direct transitions increases with application of the annealing treatment for the studied ITO samples.

**Figure 8:**
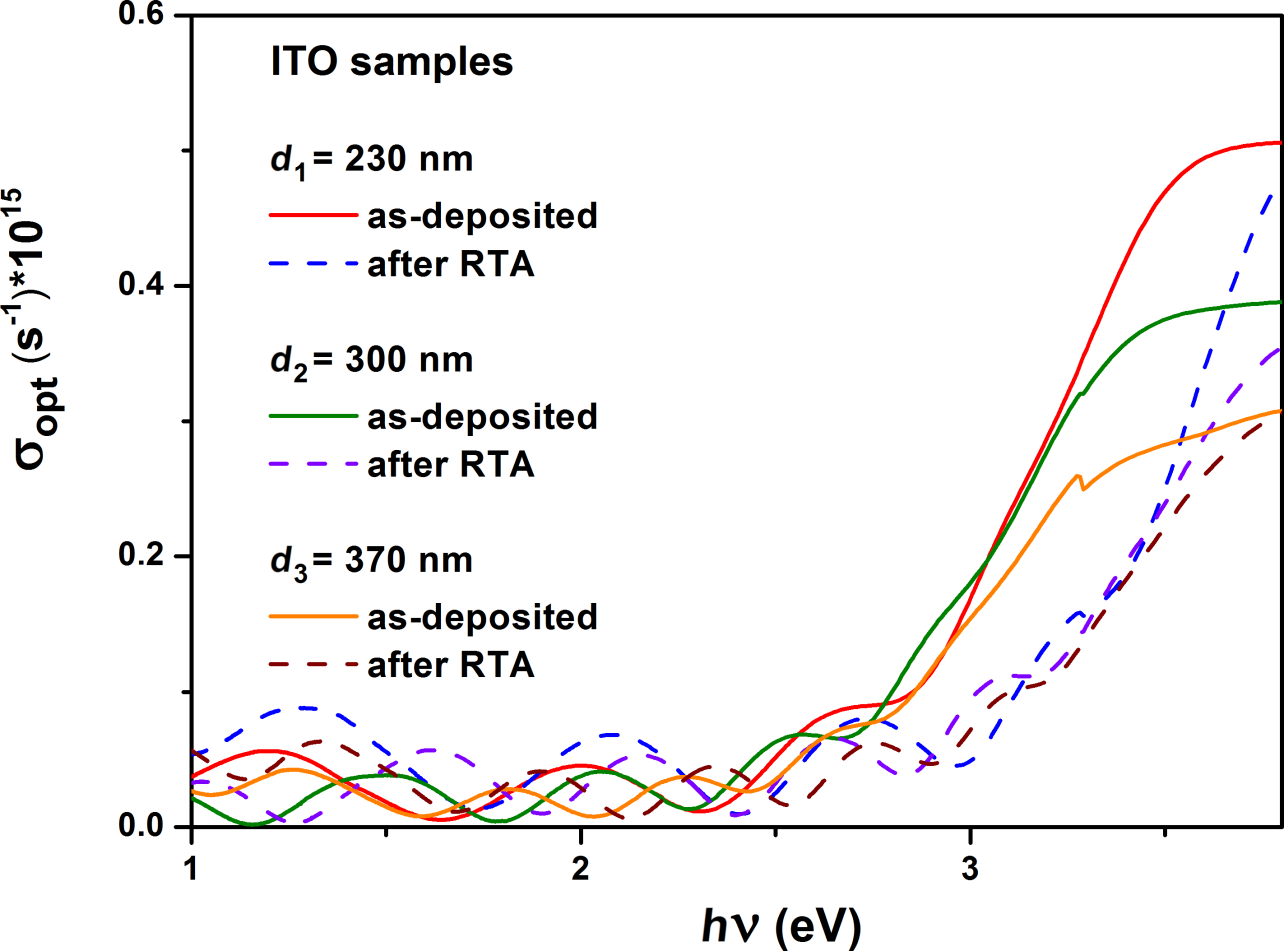
Optical conductivity dependence on the energy of the incident photons for the as-deposited and RTA-treated ITO samples.

The optical conductivity coefficient dependence (Figure 8) on wavelength is similar to that of the absorption coefficient, according to the relationship [42,45,47]:

[5]
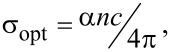


where α is the absorption coefficient, *n* is the refractive index and *c* is the speed of light.

**Figure 9:**
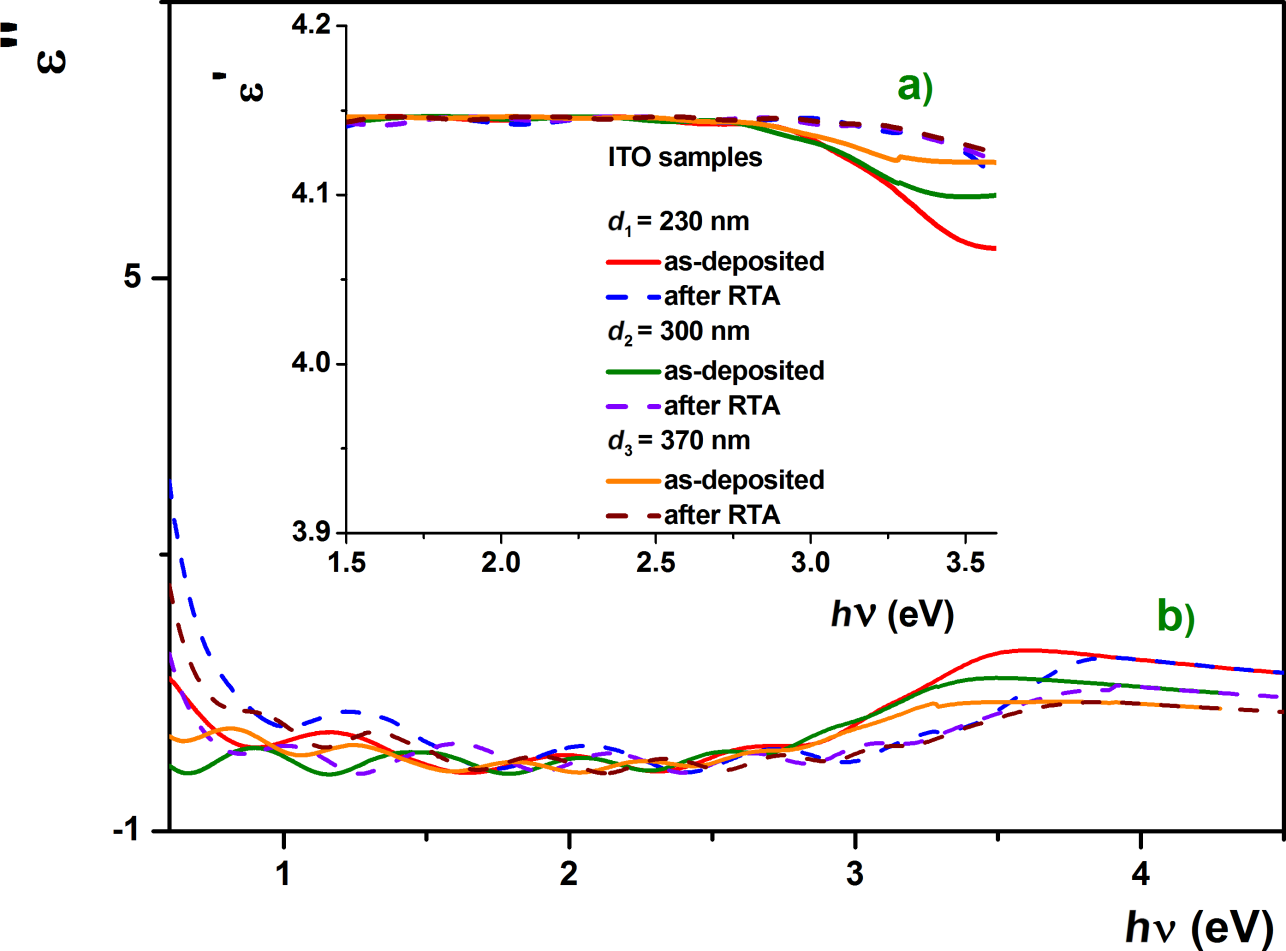
Photon energy dependence of the a) real and b) imaginary part of the complex dielectric permittivity for the as-deposited and RTA-treated ITO samples.

According to the Drude theory, depending on the dielectric parameters and concentration of carriers, *N*, the real and imaginary parts of the complex dielectric permittivity characterizes the transparency of thin films to electromagnetic radiation (see Figure 9a,b). Thus, when the imaginary part, ε'', can be neglected, the layer is transparent to electromagnetic radiation.

The dependence of the real and imaginary parts of the dielectric permittivity on the wavelength is illustrated in Figure 8. The increase in thickness of the ITO films influenced the optical constants (i.e., Drude damping coefficient, Drude frequency, complex permittivity, refractive indices, extinction coefficients), causing them to decrease.

The conductivity of the ITO thin films was determined using the four point-probe method. It was found that the electrical conductivity of the ITO film (*d* = 370 nm) increases from 5.8 × 10^3^ Ω^−1^ cm^−1^ to 1.35 × 10^4^ Ω^−1^ cm^−1^ after annealing. We may conclude that the increase in grain size and the improvement of crystallinity led to the decrease in defects in the films, thus resulting in higher conductivity. Adding oxygen leads to improved transmission and conductivity in the ITO thin films. The conductive ITO thin films (ρ = 8.4 × 10^−5^ Ω cm to ρ = 7.4 × 10^−5^ Ω cm) were obtained by RTA-treatment in an open atmosphere. The percentage of oxygen pressure used in the experiments is shown in Table 1. It is obvious that the oxygen flux during deposition affects the difference between the maximum valence band and the Fermi level. In this way oxygen-induced acceptor states are formed near the valence band (such as oxygen interstitials and indium vacancies) and the number of states increases with increasing oxygen flow. A poor crystalline quality with abundant structural defects leads to effective compensation of doping [33,48].

The metallic nature of the highly conductive ITO surface is clearly verified both by the presence of surface states and by the presence of electrons in the conduction band. A higher oxygen content (5%) was used in the deposition gases so that the Fermi level energy at the surface of ITO films was decreased, and a passivation effect of these surface states was induced.

## Conclusion

By applying the RTA process to ITO thin films deposited on quartz substrates, significant improvements to their structural, optical and electrical properties, which are necessary for photovoltaic applications, were achieved. Applying RTA resulted in improved film crystallinity by increasing the crystallite size (from 12.7 nm to 34.3 nm). Also, it was found from SEM investigations (cross-sections) that films subjected to such a thermal treatment show good adhesion to the substrate. Based on XPS investigations, it was evidenced that stoichiometric thin films were obtained. For the samples subjected to RTA, the quantity of tin oxide was found to decrease with respect to the as-deposited values.

The transmission spectra revealed that the bandgap width for the treated samples increases on average by 0.34 eV. We obtained information on dispersion of the refractive index and dielectric permittivity of the ITO films according to Wemple–DiDomenico and Drude theories, under various experimental conditions (different thicknesses), and depending on the RTA process applied after deposition. The application of this type of RTA process results in very conductive ITO electrodes with improved structural and electrical properties.
